# Case report: Maintaining altered states of consciousness over repeated ketamine infusions may be key to facilitate long-lasting antidepressant effects: some initial lessons from a personalized-dosing single-case study

**DOI:** 10.3389/fpsyt.2023.1197697

**Published:** 2023-10-25

**Authors:** Steffen Reissmann, Matthias Hartmann, Andreas Kist, Matthias E. Liechti, Kurt Stocker

**Affiliations:** ^1^Department of Psychology, University of Zurich, Zurich, Switzerland; ^2^Faculty of Psychology – UniDistance Suisse, Brig, Switzerland; ^3^Medical Office for Anesthesiology Zelenka and Colleagues, Villingen-Schwenningen, Germany; ^4^Psychopharmacology Research, Division of Clinical Pharmacology and Toxicology, Department of Clinical Research, University Hospital Basel and University of Basel, Basel, Switzerland; ^5^Chair of Cognitive Science, Department of Humanities, Social and Political Sciences, ETH Swiss Federal Institute of Technology Zurich, Zurich, Switzerland

**Keywords:** ketamine, depression, psychometrics, altered states of consciousness (ASC), 5D-ASC

## Abstract

**Background:**

The interest in psychoactive agents for treating mental disorders has gathered a growing body of scientific interest. However, research on the relationship between altered states of consciousness (ASCs) and ketamine’s antidepressant properties is still limited. Likewise, approaches to sustain early treatment success for the long-term are needed. Taking both aspects into account, the question arises whether the persistence of recurrent ASCs during the subsequent infusion sessions is crucial for the preservation of antidepressant effects during prolonged continued ketamine therapy.

**Aim:**

In this case study we explored whether recurrent ASC experiences across a large number of infusions are associated with improved antidepressant effects in a single case study.

**Methods:**

A 62-year-old patient with treatment-resistant depression, who has been suffering from depressive episodes for over 20 years, was observed for 12 consecutive infusions across 16 weeks. ASCs during ketamine sessions were measured with the 5D-ASC, and pre/post-infusion depression scores with the BDI-II questionnaire. To emphasize psychoactive experiences a personalized antidepressant dose regimen was used.

**Results:**

We found a strong correlation between the experienced ASCs during ketamine infusions and the antidepressant effect: the stronger the ASCs overall, the stronger the resulting antidepressant effect. This correlation was consistently observed throughout the infusion series, independent of the number of ketamine sessions completed before. However, despite a personalized dose regimen, neither peri-infusion ASCs nor antidepressant effects could be established on a regular basis, leading overall to no improvement in treatment outcome.

**Conclusion:**

Maintaining psychoactive effects over repeated ketamine infusions may be key to facilitate long-lasting antidepressant effects. However, for some depressed individuals maintenance of antidepressant effects and/or peri-infusion ASCs might not be achieved, even when personalized dosing is used.

## Introduction

Ever since the first report of ketamine’s antidepressant effects (1), there have also been some researchers who have attempted to bypass ketamine’s psychoactive properties (2, 3), but some degree of consciousness-alteration would generally occur (4). In this regard, the question whether ketamine’s psychoactive effects are considered adverse or viewed as an inherent component of its therapeutic potential becomes increasingly important (4, 5). A recent study by Sumner and colleagues (6) provided some first clearer, yet still preliminary, evidence for a possible role of the ASC experience in ketamine’s antidepressant properties in a crossover design with active-placebo controlled conditions and the usage of more sensitive ASC measures than previous studies. The authors reported that a greater antidepressant response correlated with specific dimensions of change in consciousness (6).

While ketamine’s general antidepressant effects are well documented, it remains yet to be shown how positive treatment effects can be sustained and turned into a long-lasting beneficial treatment outcome (7–12). In general, antidepressant effects disappear after discontinuation of the ketamine treatment and patients relapse within one to two weeks after a single infusion (8, 13, 14). Repeated ketamine infusions may prolong the antidepressant effects. In a study by Murrough and colleagues (15), the time to relapse after six infusions over twelve days averaged at 18 days. In addition, a randomized midazolam-controlled trial investigated the effect of a four-week maintenance phase with once-weekly ketamine after an initial ketamine treatment of 12 twelve days (13). This four-week maintenance period preserved antidepressant effects in 21 of 23 patients (91%) (last measurement shortly after last infusion).

However, research on possible maintenance therapies is still very limited and possible regimens on how to sustain antidepressant effects cannot be derived from the current state of knowledge. For this reason, it is of utmost importance to answer the question about how patients who respond well to ketamine therapy can further be treated to preserve the initial treatment successes. One approach that aims to incorporate ketamine’s psychoactive properties in order to sustain treatment outcomes over a longer period of time is ketamine-assisted psychotherapy (KAP) (4, 16). In this approach, consciousness-altering effects of ketamine are assumed to be an integral and necessary part of the overall therapeutic success for the treatment of a wide range of disorders, such a mood, anxiety, and addiction disorders (4). The ketamine experience is supported and integrated in a psychotherapeutic context as a “time-out” from the patient’s ordinary state of consciousness, as a relief from negativity, and as an openness to the expansiveness of mind that might include self-transcendental mystical-type qualities (4). With personalized dosing regimens (mainly sublingually or intramuscularly administered) KAP aims to achieve different states of consciousness ranging from milder trance states to fully dissociative out-of-body experiences (4). This assumes that the psychoactive effects of ketamine enhance the patient’s ability to engage in in-depth psychotherapeutic treatment and might be necessary for a beneficial long-term ketamine treatment (4). However, neither the study of Dore and colleagues (4), nor any other KAP studies we are aware of, have published any ASC data.^1^ While Sumner’s study has shown some promising preliminary results for ketamine-induced ASC-related antidepressant effects (6), a vital question has to our knowledge not been investigated empirically as of now*: What is the antidepressant potential of ketamine-induced ASCs in repeated ketamine administrations over time?*

To address this question, we report on our observations over repeated ketamine infusions in a single case for the treatment of depression where both ASC experiences and antidepressant effects were measured. This case report is part of a larger study. However, this patient was the only patient who was at the same time ketamine-naïve and received a larger number of ketamine infusions. We took advantage of this unique opportunity to see how important the maintenance of ketamine-induced ASCs over time might be in repeated-infusions ketamine therapy for depression. More specifically, we observed the experienced ASCs and antidepressant effects of a 62-year-old patient with treatment-resistant depression (22) over a series of 12 personalized-dosed ketamine infusions (16 weeks) to investigate whether (a) ASCs would generally occur in relation to the treatment, whether (b) acute ASC experiences correlate with antidepressant response, whether (c) the relationship between ASCs and antidepressant response shows a stable pattern over a long series of ketamine infusions, and if (d) specific ASC contents are associated with antidepressant effects.

ASCs were measured with a specific psychometric version of the 5-Dimensional Altered State of Consciousness Rating Scale (5D-ASC) (23, 24), which contains eleven validated subscales (42 items) (25). Depression was measured with the Beck Depression Inventory (BDI-II) (26). Furthermore, the personalized antidepressant ketamine-infusion dose regimen described in (27) was applied. In essence, in this regimen the dose level and frequency for the ketamine infusions are adjusted individually in close consultation with the patients suffering from depression with the overall goal to maximize antidepressant benefits.

## Methods

This case report is part of a larger open-label, prospective, non-interventional study authorized by the ethics committee of the Landesärztekammer Baden-Württemberg (Germany, file reference: F-2019-054) – but, as mentioned, only for this patient we were able to collect a larger number of data points (12 infusions) where the patient was at the same time ketamine-naïve at the outset of our study. The data collection was added on to an existing ketamine treatment regime of an ambulatory anesthesiologic day clinic to treat patients suffering from depression. Thus, our study design reflects the real-world setting of an outpatient ketamine infusion therapy, a setting that is becoming increasingly common (28). The patient agreed to participate in this study and provided written informed consent after the study procedures were fully explained. The patient had the (unused) possibility to withdraw from the study at any time without giving reasons and without influence on his medical treatment.

### Treatment settings

The treatment consisted of a personalized dosing regimen with an initial series of three ketamine infusions (Ketamine Inresa) within two weeks to evaluate the patient’s response to treatment, with a dosage of 0.5 mg/kg body weight and a runtime of 40 min, as this dosage/runtime has proven to be safe and has often shown antidepressant response (1, 9, 11, 29–31). After these three initial infusions, the patient’s individual response (tolerability, dissociation, and change in depressive symptomatology) determined the frequency of the subsequent infusion schedule and possible dose adaptions, to facilitate consciousness-altering effects and maximize the treatment outcome (*cf.* personalized antidepressant dose regimen (27)). Therefore, the treatment intervals were adjusted by the treating physician in close consultation with the patient. Thus, unlike in KAP (4), our personalized dosing regimen did psychoactively not go all the way to try to achieve a transformational out-of-body experience. However, the personalized antidepressant dosing regimen that we used in this study still differs significantly from the widely used medical model of ketamine therapy where ASCs are not specifically sought after (9, 11). In this dose regimen, any ASCs that feel pleasant – e.g., bodily lightness, opposite diminishing and non-judging – (27, 32) are welcome ASCs. During ketamine infusions, the patient’s vital signs were monitored by continuous electrocardiogram (ECG) monitoring, pulse oximetry, and blood pressure measurement as well as regular controls by the clinic’s physicians or medical staff.

After the end of each infusion session, the patient remained in the clinic, whereas the length of the stay was determined by the tolerability of the infusion and the patient’s physical and mental response to the medication. To capture the depth of the experienced ASCs and the severity of the depressive symptomatology, the 5D-ASC (25) was used in combination with the Beck-Depressions-Inventory (BDI-II)^2^ (26). According to literature, 40–60 min after the infusion is stopped, all consciousness-altering effects of the treatment should have subsided (29). Within this time frame and based on the assessment of the experienced physician or medical staff, the patient was asked to complete the questionnaires, first the 5D-ASC and directly afterwards the BDI-II. A total of three questionnaires were completed for each session: a first BDI-II questionnaire immediately before the treatment as a baseline measurement, followed by the 5D-ASC and a second BDI-II questionnaire after the treatment.

By comparing the measured values of the individual sessions, changes in depressive symptomatology within one session (pre/post treatment BDI-II changes) as well as over the period of the 12 treatments could be evaluated. Using this as a base, statements regarding the long-term effect of the treatment and the individual therapy success can be derived. Ultimately, the comparison of the experienced ASCs, as measured by the 5D-ASC, with the within session changes in depression severity (BDI-II depression scores) enabled us to relate the ASC aspects to the antidepressant effect of the drug.

### Assessing altered states of consciousness

To assess ASCs we utilized the latest psychometric eleven-subscale version of Dittrich’s widely used 5D-ASC questionnaire by Studerus and colleagues, which captures various ASC dimensions that typically occur during an altered state of consciousness (23–25). In the 5D-ASC, the subjective experience during the ketamine infusion is assessed retrospectively by using a visual analog scale (rated from 0–100) for each item with the lower end ‘No, not more than usual’ and the upper end ‘Yes, much more than usual’ (23–25).

**Figure 1:**
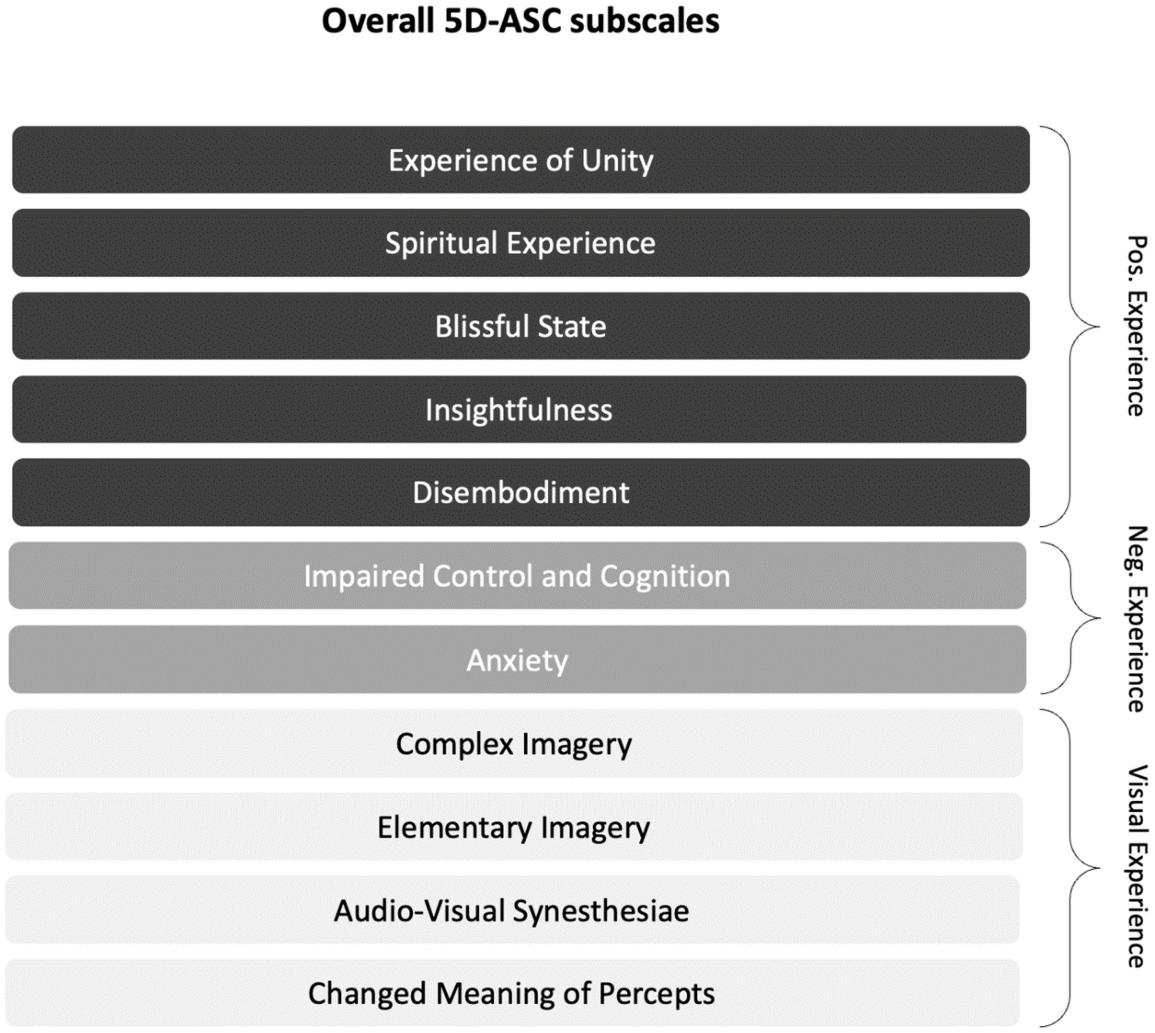
Subscales of the 5D-ASC (*cf.* (25)) and the explorative tripartite conceptual categorization thereof: The eleven validated subscales are for further exploratory purposes organized into the three conceptual categories *Positive Experience* (PE), *Negative Experience* (NE), and *Visual Experience* (VE). The conceptual categories indicate the valence of the experience (PE/NE) and the (valence-unspecific) visual aspects of the experience (VE).

Figure 1 shows the subscale structure of the 5D-ASC (25). We grouped the eleven subscales for further exploration into three conceptual categories: *Positive Experience* (PE), *Negative Experience* (NE), and *Visual Experience* (VE) (*cf.* (25, 33)). Although Studerus and colleagues (25) previously examined the higher-order categories *Pleasant Experience* and *Unpleasant Experience* as the result of hierarchical item clustering, it is important to note that PE, NE, and VE are only conceptual categories meant to guide part of our correlational ASC-antidepression analysis. The subscales of the 5D-ASC and the tripartite conceptual categorization thereof are schematically depicted in Figure 1.

### Case presentation

During a time interval of 16 weeks, from August to December 2019, a 62-year-old patient was treated with 12 repeated ketamine infusions in the day clinic. On admission, he reported that he was first diagnosed with mixed anxiety and depressive disorder in 1997, and has suffered from recurrent episodes ever since. In the year 2000, his symptoms worsened further due to financial difficulties, and in 2016 and 2017 inpatient stays followed, with a duration of ten weeks each. According to the patient’s self-report, he had undergone various psychological and pharmacological treatments over the years, including different selective serotonin reuptake inhibitors (SSRIs), serotonin noradrenalin reuptake inhibitors (SNRIs), benzodiazepines, as well as further medications which the patient did no longer recall precisely. His self-reported core symptoms consisted mainly of persistent restlessness and feelings of panic, which he also tried to relieve with alcohol in the past. He reported that previous therapies brought temporary, but no long-term improvements. Electroconvulsive therapy (ECT) had never been applied.

At the time he reached out to the clinic, he was undergoing psychotherapeutic treatment and was medicated with trazodone and pregabaline, which, as he said, also helped him to fall and stay asleep during the night. At the outset of the ketamine therapy, he showed mild depressive symptoms as measured by the BDI-II (BDI-II score = 15). In this case, an initial BDI-II score of 15 may not seem servere and many studies have included mainly patients with more pronounced depressive symptoms. However, it is important to keep in mind that besides showing mild depression symptoms, the patient has been suffering from recurrent mixed anxiety and depressive episodes of varying severity for more than 20 years without resolution.

### Data analysis

Data analyses were performed using R version 4.0.5 and Jamovi version 1.8.1.0. At first BDI-II and 5D-ASC scores were calculated as a total sum of their items separately for each session. Changes in BDI-II scores from pre- to post-infusion were given in percent. Positive values represent a reduction in BDI-II scores (improved depression symptoms). Additionally, 5D-ASC scores were calculated separately as total values for each of the elven subscales and subsequently for explorative purposes for the three overall conceptual categories (PE, NE, VE).

Statistical assessments were based on non-parametric tests to account for the small number of datapoints (n = 12 sessions). BDI-II changes over time were analyzed using the Wilcoxon signed-rank test. Kendall’s Tau rank correlations coefficient has been used to determine correlations between ASCs and depressive symptomatology (34).

## Results

### Depressive symptoms and experienced altered states of consciousness

**Figure 2:**
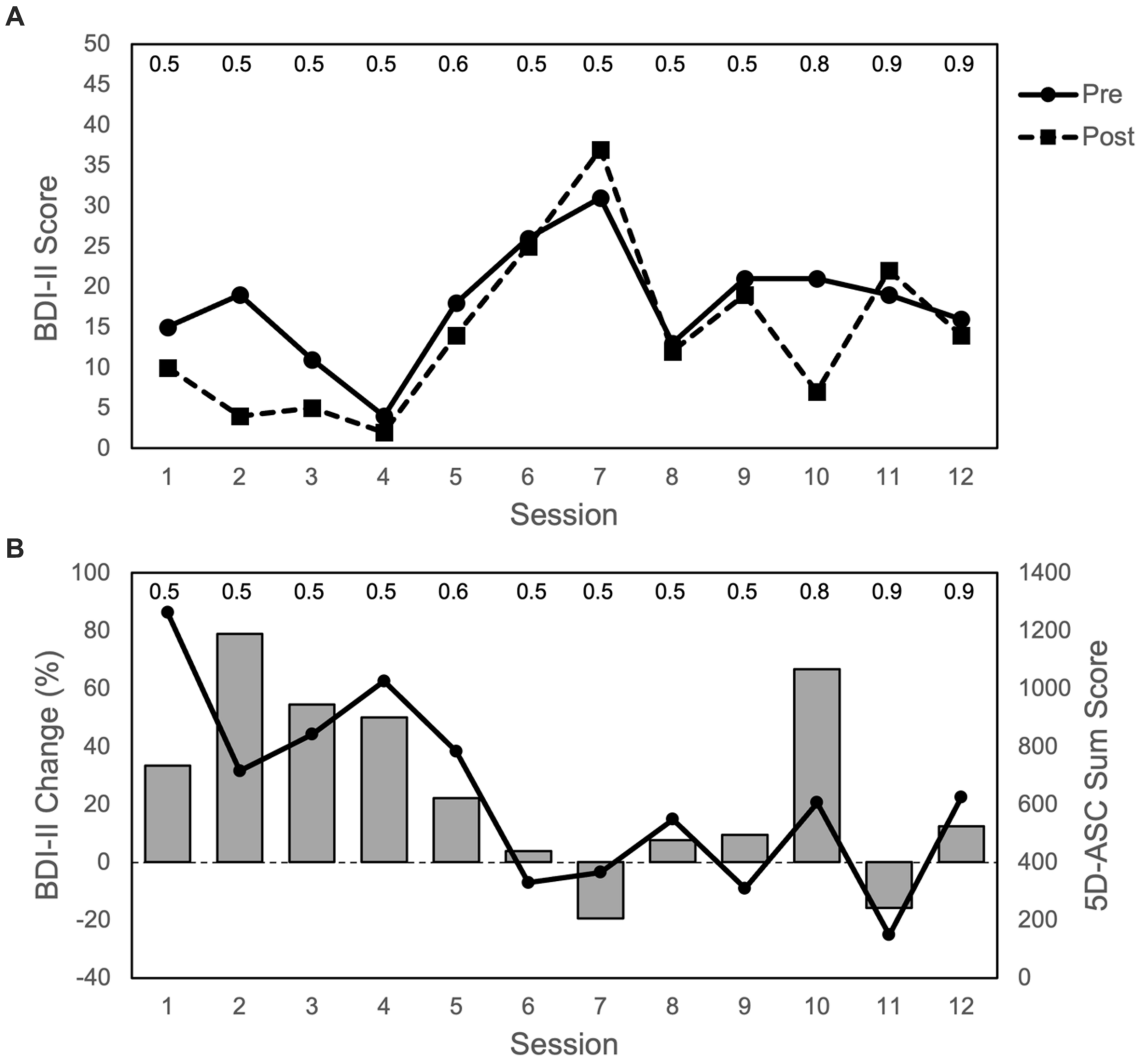
Depression and ASC scores over the course of 12 sessions: **(A)** BDI-II scores (max. = 63 points) with pre and post infusion measurements. **(B)** Bars represent changes in BDI-II scores from pre to post infusion of each session (in %); positive values represent an improvement in depressive symptoms (i.e., reduction in BDI-II scores), and negative values represent an impairment in depressive symptoms (i.e., increase in BDI-II scores). Dots and line show ASC scores (max. = 4,200 points) for 5D-ASC (42 items). Data represent single measurements from one patient. The numbers on top of the figure indicate the ketamine dose (mg/kg body weight) for each session. The duration between sessions varied between 3 and 28 days (3, 5, 7, 11, 22, 33).

All BDI-II changes reported in this section refer to the differences between BDI-II measurements pre- and post-infusion of a ketamine session, not to the changes between sessions. In his initial sessions, the patient responded well to the ketamine infusions, with major improvements up to clinically unremarkable symptom severity and a steady decrease in depression scores from infusion to infusion. The lowest score was measured after the fourth treatment with a BDI-II of 2. After his good responses in the initial period, no long-term preservation of these responses was achieved in the patient’s BDI-II scores (Figure 2). Across all 12 treatments, there was no significant antidepressant effect when comparing BDI-II scores before and after treatment (Wilcoxon W (11) = 62.5; *p* = 0.071).

During the initial phase, where the patient showed major improvements with regards to his symptom severity, the ASCs he experienced were the strongest (Figure 2). This was supported not only by the 5D-ASC measurements, but also by the patients verbal reports given after each session. After his first treatment, he described the experience during the infusion as “outstanding.” At the beginning of the first session, everything appeared dark and lightened up more and more until everything was very bright. Colors were very vibrant with a lot of yellow and blue, and he had the feeling of floating. He thought of positive things, whenever he was able to hold on to his thoughts. His body scheme changed, and he said that his fingers and toes felt quite different to how they usually do.

In the later sessions, his BDI-II scores worsened. Before his fifth session, the patient reported that he had felt significant improvements in his depressive symptoms, which subsided during the week after his fourth treatment. Half an hour after his fifth treatment, he reported that he could barely remember today’s ketamine therapy. In the following ketamine infusion sessions, his reports were mixed with different pleasant and unpleasant experiences and subsequent dose-increases (from 0.5 up to 0.9 mg/kg body weight) did not result in a sustained antidepressant effect (Figure 2). However, after the very beneficial initial sessions and subsequent mixed experiences, there was again a marked improvement in his BDI-II score in response to the tenth infusion with concomitant more pronounced ASCs.

To bring the depression and ASC measurements together: During the first four treatments, depression symptoms (measured as pre/post treatment BDI-II changes) improved while overall ASC scores were the highest. Afterwards depressive symptoms worsened while ASC scores decreased, with the one exception at the tenth treatment where depressive symptoms again improved and ASC scores again increased to some degree (Figure 2). These measurements were supported by the patient’s verbal reports at all time points.

### ASCs and antidepressant effects

To test for correlations between the experienced ASCs and possible changes in depressive symptomatology: (a) we first assessed the relationship between the 5D-ASC total scores (42-item version) and BDI-II change scores, (b) we then compared each of the 5D-ASC subscales with BDI-II change scores and (c) finally compared for explorative purposes the conceptual categories (PE, NE, VE) with the BDI-II change scores to test whether valence of experience influences antidepressant outcome.

**Figure 3:**
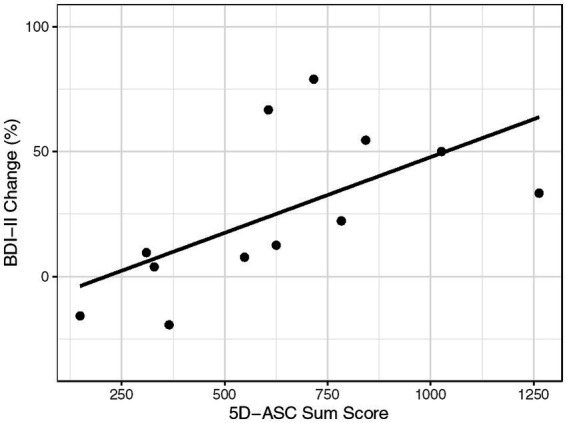
Correlation graph. BDI-II Change (in %) and 5D-ASC Sum Score for each of the 12 assessments.

The results are summarized in Table 1 and visualized in Figure 3.

- The Kendall rank correlation coefficient showed significant positive correlations between the 5D-ASC and the within session BDI-II change score, reflecting an improvement in depression symptoms with increasing ASCs (*r* = 0.485, *p* = 0.031).
- When inspecting the 11 subscales of the 5D-ASC (Table 1), *Experience of Unity, Spiritual Experience*, *Blissful State*, and *Changed Meaning of Percepts*, were significantly associated with an improvement in depressive symptoms, whereas *Anxiety* was associated with an impairment in depressive symptoms (all ps < 0.05; see Table 1).
- Similarly, the additional exploratory analysis showed that the conceptual categories *Positive Experience* and *Visual Experience* were associated with an improvement in depressive symptoms (ps < 0.05), whereas no association was found for *Negative Experience* (Table 1).

**Table 1:** Mean scores, Kendall Tau correlation coefficients and *p*-values for the association between ASCs (5D-ASC total sum, 42 items; eleven subscales; and the exploratory categories *Positive Experience*, *Negative Experience*, and *Visual Experience*) and BDI-II change (in %).

Subscale	Mean	SD	N_Items_	*r*	*p*
5D-ASC total	630.2	321.2	42	0.485	0.031
Experience of unity	91.0	86.2	5	0.443	0.046
Spiritual experience	48.0	27.1	3	0.606	0.005
Blissful STate	64.1	72.3	3	0.540	0.017
Insightfulness	14.3	27.8	3	0.431	0.066
Disembodiment	83.7	58.3	3	0.424	0.063
Impaired control and cognition	186.3	128.5	7	0.152	0.545
Anxiety	52.8	63.7	6	−0.523	0.019
Complex imagery	34.2	40.2	3	0.375	0.096
Elementary imagery	27.9	38.9	3	0.326	0.147
Audio-visual synesthesiae	7.1	11.4	3	0.348	0.140
Changed meaning of percepts	20.8	34.5	3	0.500	0.027
Positive experience	301.1	220.7	17	0.485	0.031
Negative experience	239.2	136.1	13	−0.091	0.737
Visual experience	89.9	97.8	12	0.455	0.045

Positive coefficients reflect improved depressive symptoms (i.e., decreased BDI-II scores from pre to post) with increasing ASCs. N_items_ gives the number of items within the 5D-ASC subscale. The theoretical score range for each of the questionnaire items is 0–100. Data represent values based on 12 assessments per subscale within one patient.

## Discussion

While it has already been hypothesized that dissociative symptoms of ketamine could become a clinical marker to predict treatment response (35, 36), the relationship between ASCs and ketamine’s antidepressant properties still needs to be investigated further. In this case study we found a significant relationship between the intensity of the ASC experience and the antidepressant effect. The stronger these ASCs overall, the stronger the resulting antidepressant effect. This co-occurrence between ASCs and antidepressant effects was observed both in early and late sessions, suggesting that this relationship is present over repeated sessions.

During the initial sessions, the patient showed a good response to the ketamine therapy with larger percentage changes in BDI-II reductions, while experiencing stronger ASCs. In the further course, strong fluctuations of the depression scores occurred between the sessions. A sustained stable and consistent antidepressant effect was not achieved. Even a subsequent increase in dose (up to 0.9 mg/kg body weight) failed to stablize either the ASCs experienced or the antidepressant effect. This strengthens our assumption that there is a direct positive influence in the experienced intensity of ASCs with the resulting antidepressant effect. However, our findings diverge from the results of previous studies, which demonstrated dose-dependent increases in efficacy (37) as well as dose–response relationships for psychotomimetic symptoms (38). But it is important to stress, that neither the overall duration of the treatment cycle nor the number of total ketamine sessions per patient in these studies were similarly high as in the presented case. Results from studies using other ASC-inducing agents such as psilocybin and other serotonergic psychedelics support the hypothesis that the characteristic of the acutely induced ASC effects may be associated with the long-term therapeutic effects of these rapidly acting novel antidepressants (39–41).

Investigating the associations between the 5D-ASC’s subscales and the depression scores, we found significant correlations for the subscales *Experience of Unity, Spiritual Experience*, *Blissful State* and *Changed Meaning of Percepts*. This is similar to what has been found in ketamine treatment by Sumner and colleagues (significant relationship between percentage change in depression scores and the 5D-ASC subscales *Experience of Unity*, *Spiritual Experience* and *Insightfulness*) (6).

Additionally, the explorative conceptual categories *Positive Experience* and *Visual Experience* yielded significant correlations. These results corroborate findings from classic psychedelics, which also report that such meaningful and insightful experiences are often associated with therapeutic effects (6, 41–44). Contrary to a previous case study (with two patients) (45) and Sumner and colleagues (6), which reported that negative experiences do not diminish the treatment outcome, there was no correlation between the conceptual category *Negative Experience* and the antidepressant effect in our study. Moreover, there was a negative correlation between the subscale *Anxiety* and the BDI-II scores, indicating that profound anxiety-provoking experiences did worsen the therapeutic outcome in our single case. In line with other studies, this raises the possibility that diffuse fearful ASC experiences without positive counterparts within the same experience might not be linked to positive outcomes, and that antidepressant effects also have to involve positive experiences of ASCs (36, 41, 46). Note also that the negative ASC experiences reported in the previous case study (45) and the one reported here cannot be directly compared, since the former negative ASC experience included (for both cases) a body-destruction vision, whereas the latter negative ASC experience (our case) only involved a diffuse sense of anxiety and no strong visual experience.

The growing evidence for the association between psychoactive effects and antidepressant efficacy of ketamine (4, 6, 30, 32, 36, 47) raises the question of whether ASC effects also have a major impact on the long-term maintenance of the treatment effect. In our case study, experienced ASC effects were closely linked to the antidepressant outcome at each individual season. If the patient experienced strong ASCs during the infusion, this resulted in an increased reduction of his depression scores, while the opposite was observed when the patient had an overall weaker ASC experience (see Figure 2). Independent of the number of the already completed ketamine sessions, the intensity of the experienced ASC was a good indicator for the treatment outcome of the session. This observation, although derived from a single case, gives some first evidence for guidance of future studies that ASC effects are not only crucial for the initial antidepressant effects, but that their maintenance in subsequent succession could contribute significantly to the long-term success of the therapy. In their approach of KAP, Dore and colleagues (4) already integrate ketamine-induced ASC effects into psychotherapy on a longer-term basis. We also share the believe that integrating the ketamine experience into a broader therapeutic approach is a worthwhile endeavor. ASC effects should neither be feared nor avoided, but instead consciousness-altering effects could – should future studies replicate our finding – become a beneficial part of the treatment that can be fostered with personalized antidepressant dose regimens to maximize antidepressant treatment outcomes.

## Conclusion and outlook

The findings of this case study point to the possibility that the maintenance of ASC effects over repeated ketamine infusion sessions within longer therapy cycles could be key to facilitate a long-lasting antidepressant outcome. More specifically, the initial, preliminary lessons from our ketamine single-case study are: a stronger overall ASC experience correlates with antidepressant response; this pattern – lower depression when ASCs are higher and vice versa – remains stable over time; especially positive and visual ASC experiences correlate with antidepressant response, whereas ASC anxiety experiences (without additional positive and visual ASC experiences) correlate with increase in depression; ASC experiences of brightness, vibrant colors, floating, and the occurrence of positive thoughts (that one tries to hold on to) are examples of specific ASC experiences that the patient himself associated with antidepressant response. Furthermore, our findings point to the possibility that the occurrence of antidepressant ASCs might not (or not on a long-term basis) be achieved for some individuals even when personalized dosing is used to maximize antidepressant benefits. Future randomized controlled studies are needed to investigate the influence of ketamine’s ASC properties in order to sustain antidepressant effects. This includes investigations that study this relationship across repeated ketamine infusion sessions within longer therapy cycles, and which furthermore analyze if such effects could be optimized for some individuals when dosing is personalized with the overall goal to reduce depression.

One main implication to which our case study points to is that future studies should investigate whether regular ketamine therapy is only worthwhile for individual cases, when positive and/or visual ASC effects during the ketamine experience can be maintained across sessions. If this cannot be maintained, it might be the case that a therapeutical approach other than ketamine might be indicated. Given that classic-psychedelic ASC effects have the potential of longer-term antidepressant effects even after a single administration of a classic-psychedelic substance (e.g., (48)), it is open for future investigation whether antidepressant ASCs can be occasioned more efficiently with classic psychedelics such as psilocybin than with dissociative anesthetics such as ketamine.

### Limitations

In this study, all data are based on measurements from a single individual. It is important to keep in mind that in individual cases personal circumstances and events can have a major influence on the clinical outcome. There was no control group. Furthermore, the setting of an increasingly common outpatient therapy was at the expense of controlled conditions of a clinical setting. Additionally, if still more details had been collected about this patient’s diagnostic condition and his own perspective on the therapeutic process, then we could have provided a still more differentiated overall picture of this particular case. Finally, we used the personalized dosing regimen already explained in Stocker and colleagues (27) in which the main criterion for determining the optimal personalized dose is the subjective feedback of the patient on whether she or he feels that the effect of the infusion is sufficiently strong to have an antidepressant effect. In addition to this process, no other methods were used to calibrate an optimal dosage, such as utilizing EEG measurement (*cf.* (49)). However, unlike in KAP (4), we did not have the overall goal in mind to attempt a “[r]eduction of body and sensory awareness of an ego reductive, spiritual and liberatory nature” ((4): p. 192). Thus, we do not know whether a different long-term outcome might have been achieved for the patient, if we had attempted to personalize the dose with a more psychedelic-transformational goal in mind. The applied outpatient therapeutic approach and the defined inclusion criteria were at the expense of more controlled study conditions. Lastly, this case report shares a common problem that generally exists in research with rapid-acting antidepressants (RAAD): the lack of an assessment tool that was specifically designed to capture short-term changes (within hours) in depression symptoms.

## Data availability statement

The raw data supporting the conclusions of this article will be made available by the authors, without undue reservation.

## Ethics statement

The studies involving humans were approved by Landesärztekammer Baden-Württemberg. The studies were conducted in accordance with the local legislation and institutional requirements. The participants provided their written informed consent to participate in this study. Written informed consent was obtained from the participant/patient(s) for the publication of this case report.

## Author contributions

KS and SR conceived the study. MH and SR performed statistical analysis. AK was the treating physician. SR conducted the data collection on-site and drafted the first version of the manuscript. KS and ML provided critical revisions for the manuscript. All authors contributed to the article and approved the submitted version.

## Footnotes

^1^Dore and colleagues (4) mention that the Mystical Experience Questionnaire (MEQ) (17–20) and the and Ego Dissolution Inventory (EDI) (21) was used in their study. While these two questionnaires are adequate tools to measure certain aspects of the psychedelic ASC experience, the results of these measurements are not reported upon in Dore and colleagues (4).

^2^As there is no instrument available that measures changes in depressive symptoms within such a short time period as they are induced by ketamine, we changed the acquisition period from two weeks to one hour. Therefore the BDI-II instructions have been changed as follows: “[…] pick out the one statement in each group that best describes the way you have been feeling during the last hour […].”
