# Supplementary material for: Case report: Maintaining altered states of consciousness over repeated ketamine infusions may be key to facilitate long-lasting antidepressant effects: some initial lessons from a personalized-dosing single-case study
Source: Front Psychiatry. 2023 Oct 25;14:1197697. doi: 10.3389/fpsyt.2023.1197697 (PMC10634239; doi:10.3389/fpsyt.2023.1197697)
Supplement: Supplementary file 1 [file Table_1.DOCX]

| **Session ID** | **BDI-II Pre** | **BDI-II Post** | **BDI-II Change** | **5D-ASC  Sum Score** |
| --- | --- | --- | --- | --- |
| **1** | 15 | 10 | 33,3 | 1263 |
| **2** | 19 | 4 | 78,9 | 716 |
| **3** | 11 | 5 | 54,5 | 842 |
| **4** | 4 | 2 | 50,0 | 1026 |
| **5** | 18 | 14 | 22,2 | 783 |
| **6** | 26 | 25 | 3,8 | 329 |
| **7** | 31 | 37 | -19,4 | 365 |
| **8** | 13 | 12 | 7,7 | 548 |
| **9** | 21 | 19 | 9,5 | 310 |
| **10** | 21 | 7 | 66,7 | 606 |
| **11** | 19 | 22 | -15,8 | 149 |
| **12** | 16 | 14 | 12,5 | 625 |

**Supplement 1:** BDI-II and 5D-ASC Scores for each of the 12 assessments.
